# Microscopically Controlled Surgery in the Management of a Rare Adnexal Neoplasm: A Two‐Center Experience of Eccrine Porocarcinoma Treated Through Tubingen Torte Technique

**DOI:** 10.1111/ijd.17943

**Published:** 2025-06-27

**Authors:** Federica Scarfì, Michele Lanzetti, Imma Savarese, Greta Venturi, Vittorio Berruti, Garavello Giulia, Calcinai Alessandra, Alessia Gori, Carla Cardinali, Franca Taviti

**Affiliations:** ^1^ SOC Dermatology Pistoia Prato USL Toscana Centro‐Prato Hospital Prato Italy; ^2^ Section of Dermatology Health Sciences Department, University of Florence Florence Italy; ^3^ SOC Dermatology Pistoia Prato USL Toscana Centro‐Pistoia Hospital Pistoia Italy; ^4^ SOC Pathology Unit Empoli Prato USL Toscana Centro‐San Giuseppe Hospital Empoli Italy; ^5^ SOC Pathology Unit Pistoia Pescia USL Toscana Centro, Santi Cosma e Damiano Hospital Pescia Italy

**Keywords:** adnexal tumors, eccrine porocarcinoma, Mohs surgery, Tubingen torte

1

First described by Pinkus and Mehregan in 1963, eccrine porocarcinoma (ECP) is a rare malignant tumor of adnexal lineage. Its biological behavior is marked by local aggressiveness and a high potential for distant metastasis, with the literature showing controversial evidence regarding overall survival and prognosis [1, 2]. Despite the lack of unanimous consensus on the best surgical management of ECP, micrographic surgery is acknowledged as a safe and effective procedure, offering a higher level of local disease control than wide local excision [3]. Among microscopically controlled techniques, the Tubingen torte technique (TT) represents a Mohs‐derivative protocol that meets all PDEMA (*peripheral deep en face margin assessment*) requirements defined by the American National Comprehensive Cancer Network (NCCN) guidelines. After a minimally invasive excision, both traditional Mohs and TT involve 100% margin evaluation, though they employ slightly different strategies. Traditional Mohs involves pathological sampling of the entire specimen, while in TT, only a horizontal section from the bottom of the lesion, along with a narrow marginal strip (ideally 1–3 mm), cut around the tumor edge, is sent for fresh or permanent histopathological examination. The TT margin‐strip method results in greater time savings and requires less training for the surgical team.

We present a retrospective analysis of all ECPs excised through TT at the Dermatology Unit of Santo Stefano Hospital in Prato and San Jacopo Hospital in Pistoia, Italy, between September 1, 2021, and January 1, 2024. During the investigation period, we identified 16 patients affected by ECP. All patients had a histologically confirmed primary neoplasm, except for one who had a secondary lesion with an occult primary. Of the 16 patients, seven (47%) were male, and nine (53%) were female. The mean age at diagnosis was 82 years. The most common anatomic site was the head and neck area (42%), followed by the upper limbs (26%), lower limbs (21%), and trunk (11%). None of the ECPs arose from a pre‐existing eccrine poroma. The mean size of the ECP at diagnosis was approximately 23 mm (range 8–55 mm), and the most frequent clinical presentation was a solitary nodule without ulceration (Table 1).

**TABLE 1:** Clinical, histological, and surgical characteristics of the patients (*n* = 16) included in the study.

Sex	Age	Clinical presentation	Diameter of lesion (mm)	Ulceration	IHC markers	Affected body region	No. of surgical stages	Surgical closure technique	Follow‐up (months)	Other skin tumors
M	84	Bluish nodule	35	N	CK7+, CD117+, Ki67 40%–50%	Left thigh	1	Flap	41	—
M	84	Erythematous plaque	30	N	CK7+, Ki67 30%–40%	Scalp	1	Flap	35	—
F	85	Exophytic nodule	16	Y	CK7+	Left shoulder	1	Flap	6	—
M	89	Nodule	10	Y	CK7+	Scalp	1	Skin graft	33	SCC
M	73	Erythematous plaque	30	Y	CK7+	Upper lip	2	Flap	8	—
F	82	Erythematous plaque	16	N	CK7+	Dorsal right hand	2	Skin graft	14	SCC
M	86	Nodule	8	Y	CK7+	Left leg	1	Flap	44	SCC
F	93	Nodule	15	Y	CK7+	Right glute	1	Primary closure	43	BCC
M	58	Nodule	18	N	CK7+	Scalp	1	Flap	35	—
F	91	Exophytic nodule	55	N	CK7+	Neck	1	Primary closure	3	—
F	90	Exophytic nodule	10	N	CK7+	Dorsal left hand	1	Primary closure	29	SCC
F	68	Erythematous plaque	30	N	CK7+, EMA	Abdomen	2	Flap	31	—
F	76	Erythematous plaque	17	N	CK7+, p40	Right shoulder	1	Primary closure	48	—
M	68	Nodule	10	N	CK7+	Left temple	1	Skin graft	12	BCC, SCC
F	93	Nodule	25	N	CK7+	Left cheek	1	Primary closure	6	—
F	99	Exophytic nodule	40	Y	CK7+	Right leg	2	Flap	12	—

Abbreviations: BCC, basal cell carcinoma; F, female; IHC, immunohistochemistry; M, male; SCC, squamous cell carcinoma.

**FIGURE 1:**
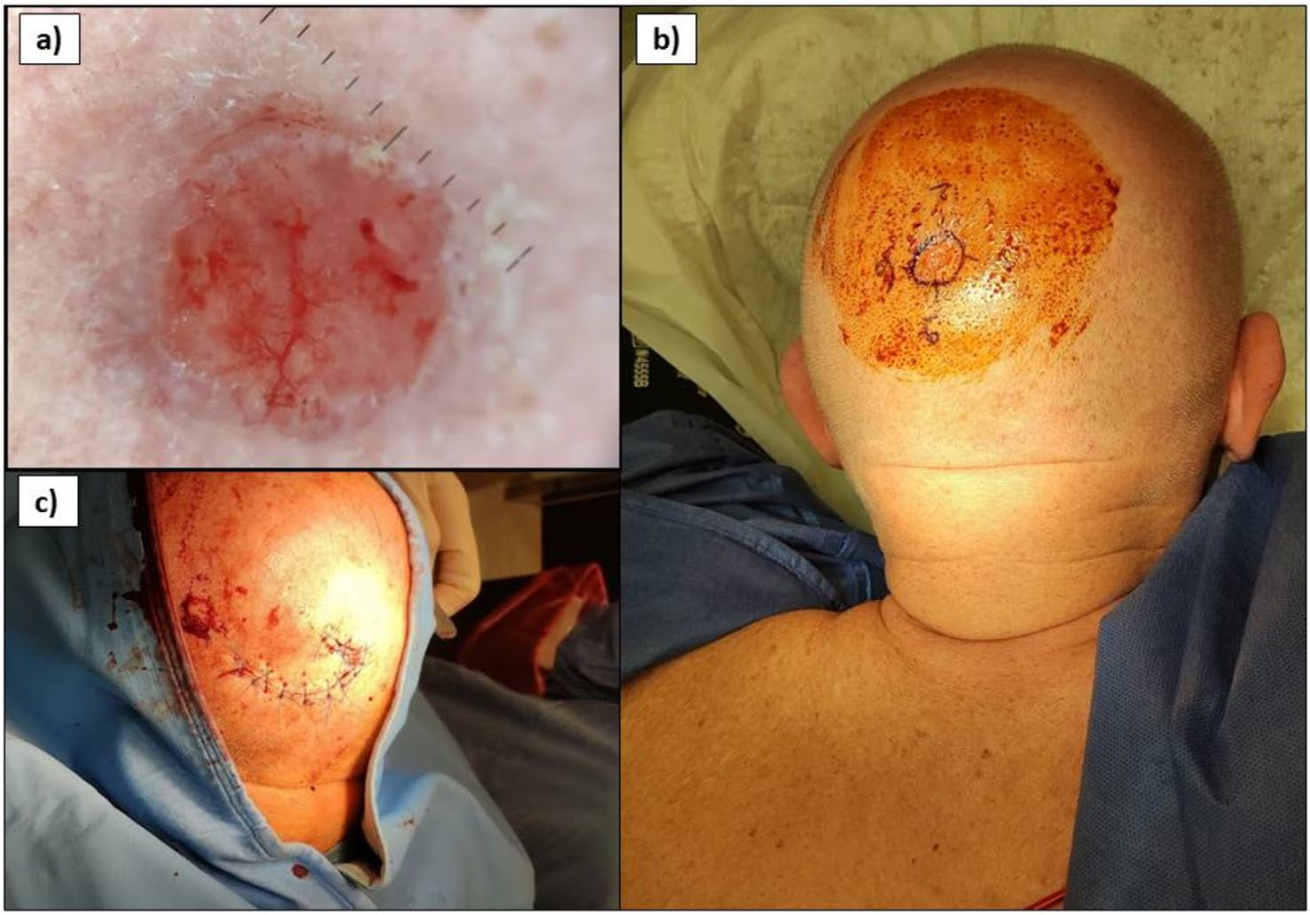
(a) Eccrine porocarcinoma (ECP) of the left temple in a 68‐year‐old male, whose dermoscopy demonstrates polymorphic vessels with milky‐red globules. Dermoscopy allowed for preoperative demarcation of the visible margins of the lesion before surgical excision. (b) ECP arising over the posterior head of a 58‐year‐old male. ECP can arise de novo or from a pre‐existing long‐standing eccrine poroma, usually located in sun‐exposed body regions, as ultraviolet (UV) exposure and immunosuppression contribute to the neoplasm's pathogenesis. The operative field shows the demarcation of the lesion and margin orientation in four circular sectors. (c) Only one surgical stage was performed in this patient, and the breach was repaired using a rotation flap.

Before the intervention, visible margins were outlined using dermoscopy (Figure 1). In the initial surgical phase, tumor removal was performed with a 90° incision angle and 5 mm margins, extending to the depth of the muscle fascia [4]. Subsequently, the specimens were sent for cryostat processing and evaluation by an on‐site pathologist. If any residual neoplastic infiltration was identified (R1), additional surgical stages were carried out until margin clearance was achieved (R0). All patients were scheduled for follow‐up evaluations every 3–4 months, as appropriate staging was recommended. The mean follow‐up period was 25 months (range 3–48), and no postoperative complications, local recurrences, lymph node, or visceral metastases were documented.

In conclusion, we describe one of the largest groups of ECP patients treated with the Tubingen torte technique, achieving recurrence rates of 0% and no distant metastases. Although these figures should be critically evaluated due to methodological drawbacks, including a small sample size and lack of randomization, our findings align with previous studies in considering micrographic surgery a cornerstone of surgical approaches for EPC. In addition to Tubingen, the torte technique incorporates the unique advantages presented by traditional Mohs, such as minimal rates of recurrence and conservative removal of the tumor in functionally constrained body regions [5], while also saving time and presenting a more feasible learning curve for the surgical team. Due to the reasons above, TT may be considered an effective alternative in managing histologically confirmed EPC when traditional Mohs micrographic surgery is unavailable.

## Consent

The patients in this manuscript have given written informed consent to participate in the study and to have their de‐identified, anonymised, aggregated data and case details (including photographs) used for publication.

## Conflicts of Interest

The authors declare no conflicts of interest.
